# Methyl *N*-(3-cyano­picolino­yl)-l-tryptophanate

**DOI:** 10.1107/S160053681303153X

**Published:** 2013-11-23

**Authors:** Olga Ovdiichuk, Olga Hordiyenko, Zoia Voitenko, Axelle Arrault, Volodymyr Medviediev

**Affiliations:** a Kyiv National Taras Shevchenko University, Department of Chemistry, Volodymyrska str. 64, 01601 Kyiv, Ukraine; b Laboratoire de Chimie Physique Macromoleculaire, UMR 7568, ENSIC, BP 451, 54001 Nancy, France; c STC "Institute for Single Crystals", National Academy of Science of Ukraine, Lenina ave. 60, 61001, Khar’kov, Ukraine

## Abstract

In the title compound, C_19_H_16_N_4_O_3_, the stereocenter has an l configuration; l-tryptophan methyl ester hydro­chloride being used as a starting material. The indole ring system and the pyridine ring are inclined to one another by 13.55 (14)°. In the crystal, adjacent mol­ecules are linked *via* N—H⋯O hydrogen bonds, forming chains propagating along the *c*-axis direction.

## Related literature

Cyano-substituted compounds, like the title compound, are useful as inter­mediates in the synthesis of *N*-hy­droxy­benzamidines, see: Peterlin-Mašič & Kikelj (2001 ▶). For the synthesis of the title compound, see: Devillers *et al.* (2002 ▶). For the biological activity of 1,2,4-oxa­diazole derivatives, see: Kundu *et al.* (2012 ▶); Sakamoto *et al.* (2007 ▶); Tyrkov & Sukhenko (2004 ▶).
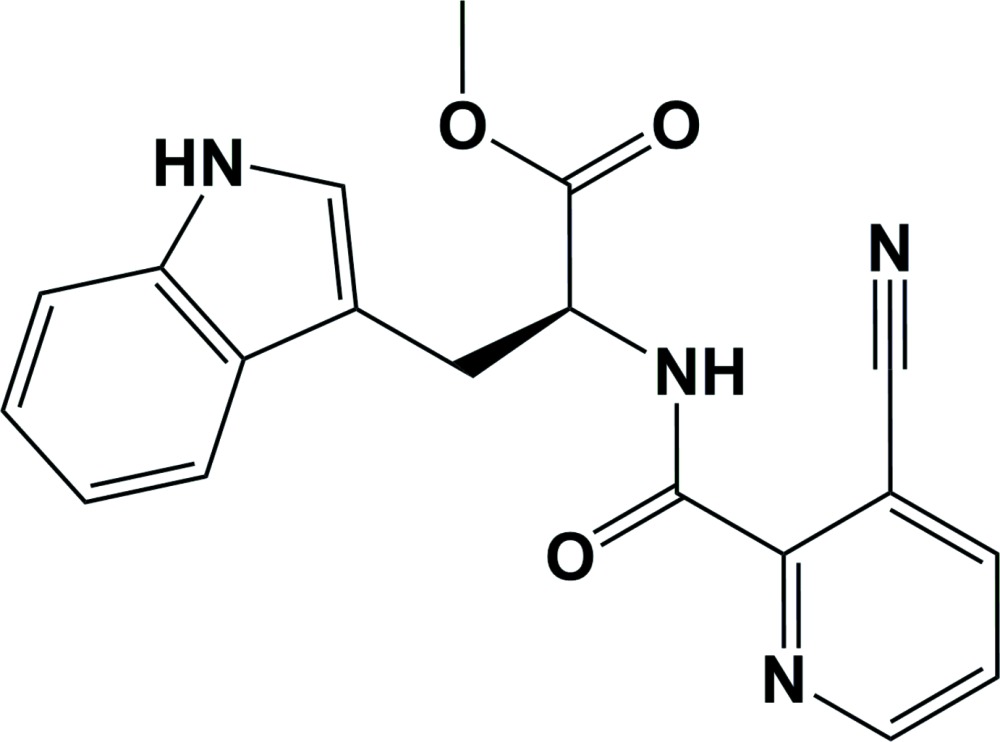


## Experimental

### 

#### Crystal data

C_19_H_16_N_4_O_3_
*M* *_r_* = 348.36
Monoclinic, 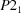

*a* = 7.473 (2) Å
*b* = 11.977 (4) Å
*c* = 9.661 (3) Å
β = 91.01 (2)°
*V* = 864.6 (4) Å^3^
*Z* = 2
Mo *K*α radiation
μ = 0.09 mm^−1^
*T* = 293 K
0.34 × 0.29 × 0.21 mm

#### Data collection

Agilent Xcalibur Sapphire3 diffractometer
Absorption correction: multi-scan (*CrysAlis PRO*; Agilent, 2011 ▶) *T*_min_ = 0.753, *T*_max_ = 1.000
9857 measured reflections
4832 independent reflections
2596 reflections with *I* > 2σ(*I*)
*R*_int_ = 0.047

#### Refinement

*R*[*F* ^2^ > 2σ(*F* ^2^)] = 0.051
*wR*(*F* ^2^) = 0.133
*S* = 0.93
4832 reflections
236 parameters
1 restraint
H-atom parameters constrained
Δρ_max_ = 0.20 e Å^−3^
Δρ_min_ = −0.14 e Å^−3^
Absolute structure: Flack parameter determined using 855 quotients [(*I* ^+^)−(*I* ^−^)]/[(*I* ^+^)+(*I* ^−^)] (Parsons *et al.*, 2013 ▶)
Absolute structure parameter: −0.001 (3)

### 

Data collection: *CrysAlis PRO* (Agilent, 2011 ▶); cell refinement: *CrysAlis PRO*; data reduction: *CrysAlis PRO*; program(s) used to solve structure: *SHELXS2013* (Sheldrick, 2008 ▶); program(s) used to refine structure: *SHELXL2013* (Sheldrick, 2008 ▶); molecular graphics: *OLEX2* (Dolomanov *et al.*, 2009 ▶); software used to prepare material for publication: *OLEX2*.

## Supplementary Material

Crystal structure: contains datablock(s) I. DOI: 10.1107/S160053681303153X/su2666sup1.cif

Structure factors: contains datablock(s) I. DOI: 10.1107/S160053681303153X/su2666Isup2.hkl

Supplementary material file. DOI: 10.1107/S160053681303153X/su2666Isup3.cdx

Supplementary material file. DOI: 10.1107/S160053681303153X/su2666Isup4.cml

Additional supplementary materials:  crystallographic information; 3D view; checkCIF report

## Figures and Tables

**Table 1:** Hydrogen-bond geometry (Å, °)

*D*—H⋯*A*	*D*—H	H⋯*A*	*D*⋯*A*	*D*—H⋯*A*
N4—H4⋯O1^i^	0.86	2.29	2.987 (3)	138

Symmetry code: (i) 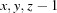.

**Fig. 1.**
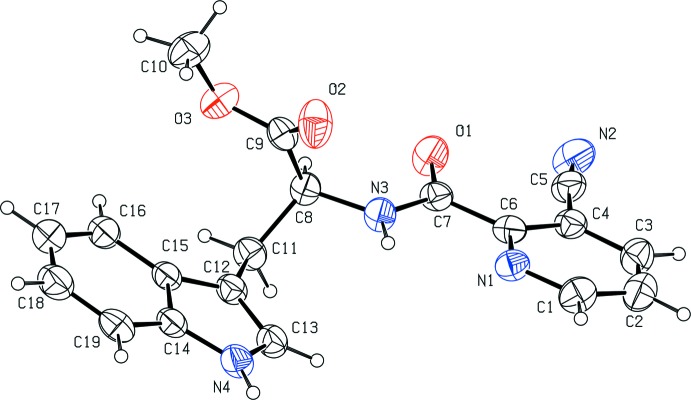
The molecular structure of the title compound, with atom labelling. Displacement ellipsoids are drawn at the 30% probability level.

## supplementary crystallographic information

### 1. Comment

Cyano substituted compounds like the title compound are useful as intermediates in the synthesis of N-hydroxybenzamidines (Peterlin-Mašič & Kikelj, 2001). Substituted *N*-hydroxybenzamidines as well as their heterocyclic analogs are key intermediates in the synthesis of pharmaceutically important derivatives of 1,2,4-oxadiazole. The latter are well known for their anticancer (Kundu *et al.*, 2012), anti-HIV (Sakamoto *et al.*, 2007), and anti-microbial activities (Tyrkov & Sukhenko, 2004). In our studies on *N*-hydroxyamidines, of a heterocyclic nature from corresponding cyano derivatives, we synthesized the title compound and report herein on its crystal structure.

The molecular structure of the title compound is illustrated in Fig. 1. The stereo center, C8, has an *L*-configuration similar to the starting material *L*-tryptophan methyl ester hydrochloride. The dihedral angle between the indole ring system (N4/C12-C19; maximum deviation 0.033 (3) Å for atom C15) and pyridine ring (N1/C1-C4/C6) is 13.55 (14)°.

In the crystal, adjacent molecules are linked *via* N—H···O hydrogen bonds, forming chains propagating along the *c* axis direction (Table 1).

### 2. Experimental

The title compound was synthesized according to the literature procedure (Devillers *et al.*, 2002). 2-Cyanonicotinic acid (5 mmol) was dissolved in CH_2_Cl_2_ (30 ml), then triethylamine (10 mmol), *L*-tryptophan methyl ester hydrochloride (5 mmol) and *N*-hydroxybenzotriazole (5 mmol) were added to the solution. The mixture was stirred at 273 K and EDCI (5.05 mmol; 1-Ethyl-3-(3-dimethylaminopropyl)carbodiimide hydroiodide) was added. Then, the mixture was stirred at room temperature over night. The residue was diluted in CH_2_Cl_2_, washed with a solution of 0.1*M* HCl (3 × 15 ml), brine (20 ml) and then dried over MgSO_4_ and concentrated under vacuo. The solid obtained was purified by column chromatography (CH_2_Cl_2_:MeOH 94:6). The title compound is a byproduct and crystallized as pale-yellow block-like crystals, suitable for X-ray diffraction analysis, by slow evaporation of a solution in dichloromethane and methanol (9:1).

### 3. Refinement

All H atoms were placed in idealized positions and constrained to ride on their parent atoms: N-H = 0.86 Å, C-H = 0.93, 0.98, 0.97 and 0.96 Å for H(aromatic), methine, methylene and methyl H atoms, respectively, with *U*_iso_ = 1.5U_eq_(C-methyl) and = 1.2U_eq_(N,C) for other H atoms.

### Crystal data

**Table:**

C_19_H_16_N_4_O_3_	*F*(000) = 364
*M**_r_* = 348.36	*D*_x_ = 1.338 Mg m^−^^3^
Monoclinic, *P*2_1_	Mo *K*α radiation, λ = 0.7107 Å
Hall symbol: P 2yb	Cell parameters from 1601 reflections
*a* = 7.473 (2) Å	θ = 3.2–32.1°
*b* = 11.977 (4) Å	µ = 0.09 mm^−^^1^
*c* = 9.661 (3) Å	*T* = 293 K
β = 91.01 (2)°	Block, colourless
*V* = 864.6 (4) Å^3^	0.34 × 0.29 × 0.21 mm
*Z* = 2	

### Data collection

**Table:**

Agilent Xcalibur Sapphire3 diffractometer	4832 independent reflections
Radiation source: Enhance (Mo) X-ray Source	2596 reflections with *I* > 2σ(*I*)
Graphite monochromator	*R*_int_ = 0.047
Detector resolution: 16.1827 pixels mm^-1^	θ_max_ = 30.0°, θ_min_ = 3.2°
ω scans	*h* = −10→10
Absorption correction: multi-scan (*CrysAlis PRO*; Agilent, 2011)	*k* = −16→16
*T*_min_ = 0.753, *T*_max_ = 1.000	*l* = −13→13
9857 measured reflections	

### Refinement

**Table:**

Refinement on *F*^2^	Secondary atom site location: difference Fourier map
Least-squares matrix: full	Hydrogen site location: inferred from neighbouring sites
*R*[*F*^2^ > 2σ(*F*^2^)] = 0.051	H-atom parameters constrained
*wR*(*F*^2^) = 0.133	*w* = 1/[σ^2^(*F*_o_^2^) + (0.0481*P*)^2^] where *P* = (*F*_o_^2^ + 2*F*_c_^2^)/3
*S* = 0.93	(Δ/σ)_max_ = 0.002
4832 reflections	Δρ_max_ = 0.20 e Å^−^^3^
236 parameters	Δρ_min_ = −0.14 e Å^−^^3^
1 restraint	Absolute structure: Flack parameter determined using 855 quotients [(*I*^+^)-(*I*^-^)]/[(*I*^+^)+(*I*^-^)] (Parsons *et al.*, 2013)
64 constraints	Absolute structure parameter: −0.001 (3)
Primary atom site location: structure-invariant direct methods	

### Special details

**Table:**

Geometry. All e.s.d.'s (except the e.s.d. in the dihedral angle between two l.s. planes) are estimated using the full covariance matrix. The cell e.s.d.'s are taken into account individually in the estimation of e.s.d.'s in distances, angles and torsion angles; correlations between e.s.d.'s in cell parameters are only used when they are defined by crystal symmetry. An approximate (isotropic) treatment of cell e.s.d.'s is used for estimating e.s.d.'s involving l.s. planes.

### Fractional atomic coordinates and isotropic or equivalent isotropic displacement parameters (Å^2^)

**Table:**

	*x*	*y*	*z*	*U*_iso_*/*U*_eq_	
O1	0.3366 (4)	0.9180 (2)	0.4295 (2)	0.0820 (8)	
O2	−0.0128 (4)	1.0476 (3)	0.1272 (4)	0.0933 (9)	
O3	0.1275 (3)	1.2086 (2)	0.1722 (3)	0.0744 (8)	
N1	0.2235 (4)	0.7036 (2)	0.1931 (3)	0.0593 (7)	
N2	0.4469 (6)	0.7467 (3)	0.6623 (4)	0.0918 (12)	
N3	0.2808 (4)	0.9245 (2)	0.2012 (3)	0.0588 (7)	
H3	0.2553	0.8865	0.1280	0.071*	
N4	0.3014 (4)	0.9839 (3)	−0.2741 (3)	0.0649 (8)	
H4	0.2870	0.9343	−0.3376	0.078*	
C1	0.2040 (5)	0.5917 (3)	0.1847 (4)	0.0733 (11)	
H1	0.1587	0.5615	0.1026	0.088*	
C2	0.2477 (5)	0.5201 (3)	0.2913 (5)	0.0779 (12)	
H2	0.2344	0.4434	0.2802	0.094*	
C3	0.3103 (5)	0.5626 (4)	0.4126 (4)	0.0710 (10)	
H3A	0.3404	0.5156	0.4860	0.085*	
C4	0.3291 (4)	0.6783 (3)	0.4258 (3)	0.0565 (9)	
C5	0.3958 (5)	0.7217 (3)	0.5548 (4)	0.0692 (10)	
C6	0.2835 (4)	0.7458 (3)	0.3133 (3)	0.0540 (8)	
C7	0.3022 (5)	0.8708 (3)	0.3205 (3)	0.0544 (8)	
C8	0.2987 (4)	1.0447 (3)	0.1894 (3)	0.0534 (8)	
H8	0.3446	1.0731	0.2783	0.064*	
C9	0.1191 (5)	1.0983 (3)	0.1613 (3)	0.0576 (9)	
C10	−0.0323 (6)	1.2707 (4)	0.1397 (6)	0.0924 (15)	
H10A	−0.0786	1.2479	0.0508	0.139*	
H10B	−0.0049	1.3490	0.1377	0.139*	
H10C	−0.1201	1.2567	0.2089	0.139*	
C11	0.4336 (5)	1.0773 (3)	0.0766 (3)	0.0611 (9)	
H11A	0.4658	1.1551	0.0895	0.073*	
H11B	0.5414	1.0333	0.0906	0.073*	
C12	0.3694 (4)	1.0617 (3)	−0.0696 (3)	0.0555 (8)	
C13	0.3676 (5)	0.9647 (3)	−0.1435 (3)	0.0628 (9)	
H13	0.4058	0.8957	−0.1100	0.075*	
C14	0.2617 (5)	1.0954 (3)	−0.2872 (3)	0.0558 (9)	
C15	0.3002 (4)	1.1473 (3)	−0.1602 (3)	0.0521 (8)	
C16	0.2762 (5)	1.2624 (3)	−0.1484 (4)	0.0651 (10)	
H16	0.3016	1.2987	−0.0653	0.078*	
C17	0.2146 (6)	1.3211 (3)	−0.2616 (5)	0.0776 (12)	
H17	0.1990	1.3979	−0.2546	0.093*	
C18	0.1748 (5)	1.2681 (4)	−0.3863 (5)	0.0800 (12)	
H18	0.1324	1.3099	−0.4610	0.096*	
C19	0.1972 (5)	1.1552 (4)	−0.4010 (3)	0.0680 (10)	
H19	0.1701	1.1197	−0.4843	0.082*	

### Atomic displacement parameters (Å^2^)

**Table:**

	*U*^11^	*U*^22^	*U*^33^	*U*^12^	*U*^13^	*U*^23^
O1	0.138 (2)	0.0690 (16)	0.0390 (13)	0.0108 (16)	−0.0117 (14)	−0.0018 (12)
O2	0.0744 (18)	0.0795 (19)	0.125 (3)	−0.0159 (16)	−0.0208 (17)	0.0269 (18)
O3	0.0749 (18)	0.0649 (16)	0.0831 (18)	0.0101 (13)	−0.0101 (13)	−0.0138 (13)
N1	0.0617 (18)	0.0628 (18)	0.0533 (18)	0.0036 (14)	−0.0028 (14)	−0.0073 (14)
N2	0.136 (3)	0.090 (2)	0.0489 (19)	0.045 (2)	−0.0133 (19)	0.0005 (18)
N3	0.0815 (19)	0.0571 (17)	0.0374 (15)	−0.0026 (15)	−0.0072 (13)	−0.0031 (12)
N4	0.088 (2)	0.0628 (18)	0.0445 (17)	−0.0164 (16)	0.0091 (14)	−0.0101 (14)
C1	0.074 (3)	0.071 (3)	0.074 (3)	−0.003 (2)	−0.008 (2)	−0.014 (2)
C2	0.075 (3)	0.060 (2)	0.099 (4)	−0.0003 (19)	−0.003 (2)	0.002 (2)
C3	0.072 (2)	0.067 (2)	0.074 (3)	0.010 (2)	−0.004 (2)	0.012 (2)
C4	0.0557 (19)	0.063 (2)	0.051 (2)	0.0115 (15)	0.0030 (15)	0.0044 (16)
C5	0.087 (3)	0.066 (2)	0.054 (2)	0.026 (2)	0.0042 (19)	0.0076 (19)
C6	0.0508 (19)	0.064 (2)	0.0478 (19)	0.0073 (16)	0.0032 (14)	−0.0031 (16)
C7	0.063 (2)	0.064 (2)	0.0362 (18)	0.0110 (16)	−0.0013 (15)	0.0013 (15)
C8	0.069 (2)	0.0526 (19)	0.0380 (16)	−0.0012 (16)	−0.0088 (14)	−0.0014 (15)
C9	0.061 (2)	0.065 (2)	0.0465 (18)	−0.0050 (17)	0.0004 (15)	0.0072 (16)
C10	0.079 (3)	0.084 (3)	0.114 (4)	0.028 (2)	−0.002 (2)	−0.002 (3)
C11	0.064 (2)	0.066 (2)	0.0531 (19)	−0.0022 (18)	−0.0010 (15)	0.0000 (17)
C12	0.0613 (19)	0.0580 (19)	0.0474 (17)	−0.0095 (17)	0.0079 (14)	−0.0039 (16)
C13	0.079 (2)	0.058 (2)	0.051 (2)	−0.0090 (18)	0.0086 (17)	−0.0012 (17)
C14	0.060 (2)	0.061 (2)	0.0473 (18)	−0.0087 (16)	0.0130 (15)	0.0006 (16)
C15	0.0559 (18)	0.0554 (19)	0.0454 (17)	−0.0082 (16)	0.0117 (14)	−0.0022 (16)
C16	0.073 (2)	0.061 (2)	0.062 (2)	−0.0071 (18)	0.0122 (18)	−0.0032 (19)
C17	0.080 (3)	0.067 (2)	0.087 (3)	0.003 (2)	0.021 (2)	0.005 (2)
C18	0.080 (3)	0.092 (3)	0.069 (3)	0.009 (2)	0.015 (2)	0.018 (2)
C19	0.071 (2)	0.088 (3)	0.0456 (19)	−0.007 (2)	0.0086 (16)	0.0029 (19)

### Geometric parameters (Å, º)

**Table:**

O1—C7	1.219 (4)	C8—H8	0.9800
O2—C9	1.199 (4)	C8—C9	1.507 (5)
O3—C9	1.327 (4)	C8—C11	1.547 (4)
O3—C10	1.437 (5)	C10—H10A	0.9600
N1—C1	1.351 (5)	C10—H10B	0.9600
N1—C6	1.337 (4)	C10—H10C	0.9600
N2—C5	1.141 (5)	C11—H11A	0.9700
N3—H3	0.8600	C11—H11B	0.9700
N3—C7	1.327 (4)	C11—C12	1.495 (5)
N3—C8	1.451 (4)	C12—C13	1.364 (5)
N4—H4	0.8600	C12—C15	1.438 (5)
N4—C13	1.366 (4)	C13—H13	0.9300
N4—C14	1.374 (5)	C14—C15	1.401 (4)
C1—H1	0.9300	C14—C19	1.391 (5)
C1—C2	1.375 (6)	C15—C16	1.394 (5)
C2—H2	0.9300	C16—H16	0.9300
C2—C3	1.354 (6)	C16—C17	1.372 (6)
C3—H3A	0.9300	C17—H17	0.9300
C3—C4	1.399 (6)	C17—C18	1.389 (6)
C4—C5	1.431 (5)	C18—H18	0.9300
C4—C6	1.392 (5)	C18—C19	1.370 (6)
C6—C7	1.505 (5)	C19—H19	0.9300
			
C9—O3—C10	117.4 (3)	O3—C10—H10A	109.5
C6—N1—C1	117.5 (3)	O3—C10—H10B	109.5
C7—N3—H3	118.7	O3—C10—H10C	109.5
C7—N3—C8	122.7 (3)	H10A—C10—H10B	109.5
C8—N3—H3	118.7	H10A—C10—H10C	109.5
C13—N4—H4	125.6	H10B—C10—H10C	109.5
C13—N4—C14	108.9 (3)	C8—C11—H11A	108.4
C14—N4—H4	125.6	C8—C11—H11B	108.4
N1—C1—H1	118.3	H11A—C11—H11B	107.4
N1—C1—C2	123.4 (4)	C12—C11—C8	115.6 (3)
C2—C1—H1	118.3	C12—C11—H11A	108.4
C1—C2—H2	120.4	C12—C11—H11B	108.4
C3—C2—C1	119.2 (4)	C13—C12—C11	126.9 (3)
C3—C2—H2	120.4	C13—C12—C15	106.8 (3)
C2—C3—H3A	120.5	C15—C12—C11	126.3 (3)
C2—C3—C4	119.0 (4)	N4—C13—H13	125.1
C4—C3—H3A	120.5	C12—C13—N4	109.9 (3)
C3—C4—C5	118.1 (3)	C12—C13—H13	125.1
C6—C4—C3	118.7 (3)	N4—C14—C15	108.0 (3)
C6—C4—C5	123.1 (3)	N4—C14—C19	130.1 (3)
N2—C5—C4	173.8 (4)	C19—C14—C15	121.8 (3)
N1—C6—C4	122.2 (3)	C14—C15—C12	106.4 (3)
N1—C6—C7	116.5 (3)	C16—C15—C12	134.5 (3)
C4—C6—C7	121.3 (3)	C16—C15—C14	119.0 (3)
O1—C7—N3	123.1 (3)	C15—C16—H16	120.5
O1—C7—C6	121.3 (3)	C17—C16—C15	118.9 (4)
N3—C7—C6	115.6 (3)	C17—C16—H16	120.5
N3—C8—H8	107.9	C16—C17—H17	119.3
N3—C8—C9	110.7 (3)	C16—C17—C18	121.3 (4)
N3—C8—C11	111.6 (3)	C18—C17—H17	119.3
C9—C8—H8	107.9	C17—C18—H18	119.5
C9—C8—C11	110.8 (3)	C19—C18—C17	121.1 (4)
C11—C8—H8	107.9	C19—C18—H18	119.5
O2—C9—O3	124.3 (3)	C14—C19—H19	121.1
O2—C9—C8	124.0 (3)	C18—C19—C14	117.8 (4)
O3—C9—C8	111.6 (3)	C18—C19—H19	121.1
			
N1—C1—C2—C3	1.4 (6)	C8—C11—C12—C13	−81.6 (4)
N1—C6—C7—O1	171.7 (3)	C8—C11—C12—C15	99.6 (4)
N1—C6—C7—N3	−8.9 (4)	C9—C8—C11—C12	−50.2 (4)
N3—C8—C9—O2	−13.3 (5)	C10—O3—C9—O2	−0.6 (6)
N3—C8—C9—O3	169.9 (3)	C10—O3—C9—C8	176.3 (3)
N3—C8—C11—C12	73.6 (4)	C11—C8—C9—O2	111.1 (4)
N4—C14—C15—C12	1.3 (3)	C11—C8—C9—O3	−65.8 (4)
N4—C14—C15—C16	178.0 (3)	C11—C12—C13—N4	−179.2 (3)
N4—C14—C19—C18	−177.9 (3)	C11—C12—C15—C14	178.4 (3)
C1—N1—C6—C4	1.5 (5)	C11—C12—C15—C16	2.4 (6)
C1—N1—C6—C7	−179.4 (3)	C12—C15—C16—C17	176.0 (3)
C1—C2—C3—C4	−0.1 (6)	C13—N4—C14—C15	−1.4 (4)
C2—C3—C4—C5	−179.9 (3)	C13—N4—C14—C19	177.7 (3)
C2—C3—C4—C6	−0.3 (5)	C13—C12—C15—C14	−0.7 (3)
C3—C4—C6—N1	−0.4 (5)	C13—C12—C15—C16	−176.6 (4)
C3—C4—C6—C7	−179.4 (3)	C14—N4—C13—C12	1.0 (4)
C4—C6—C7—O1	−9.2 (5)	C14—C15—C16—C17	0.4 (5)
C4—C6—C7—N3	170.1 (3)	C15—C12—C13—N4	−0.2 (4)
C5—C4—C6—N1	179.2 (3)	C15—C14—C19—C18	1.2 (5)
C5—C4—C6—C7	0.1 (5)	C15—C16—C17—C18	0.4 (6)
C6—N1—C1—C2	−2.0 (6)	C16—C17—C18—C19	−0.5 (6)
C7—N3—C8—C9	−109.7 (4)	C17—C18—C19—C14	−0.3 (5)
C7—N3—C8—C11	126.5 (3)	C19—C14—C15—C12	−178.0 (3)
C8—N3—C7—O1	0.8 (6)	C19—C14—C15—C16	−1.2 (5)
C8—N3—C7—C6	−178.5 (3)		

### Hydrogen-bond geometry (Å, º)

**Table:**

*D*—H···*A*	*D*—H	H···*A*	*D*···*A*	*D*—H···*A*
N4—H4···O1^i^	0.86	2.29	2.987 (3)	138

Symmetry code: (i) *x*, *y*, *z*−1.
